# The Impact of Artificial Intelligence on Financial Systems in Healthcare: A Systematic Review of Economic Evaluation Studies

**DOI:** 10.7759/cureus.86279

**Published:** 2025-06-18

**Authors:** Shyamsunder Kakatum Rao, Prashant Gupta, Asadullah Mohammed, Kanwarjit Zakhmi, Manas Ranjan Mohanty, Priji Prasad Jalaja

**Affiliations:** 1 Software Engineering, RouteOne LLC, Canton, USA; 2 Department of Technology, Intuit, Plano, USA; 3 Technical Program Manager, Kforce Inc., Houston, Texas, USA; 4 Technical Operations Manager, AWS, Portland, Oregon, USA; 5 Amazon Core Search Team, Amazon Artificial General Intelligence (AGI), Sunnyvale, USA; 6 Department of Surgery, Emory University Atlanta, Atlanta, USA

**Keywords:** artificial intelligence, cost-effectiveness, financial impact, healthcare economics, machine learning, systematic review

## Abstract

The integration of artificial intelligence (AI) into healthcare systems has emerged as a transformative approach to addressing rising costs and inefficiencies. While AI applications show promise in improving financial outcomes, the evidence remains fragmented due to methodological heterogeneity and inconsistent reporting. This systematic review aims to synthesize economic evaluations of AI in healthcare, assessing its impact on cost savings, efficiency gains, and cost-effectiveness while identifying gaps in the current literature. Following PRISMA 2020 guidelines, we conducted a systematic search across five databases (PubMed/MEDLINE, Embase, Scopus, Web of Science, and EconLit), identifying 341 records. After removing duplicates and screening for eligibility, six studies met the inclusion criteria, which focused on AI-driven economic evaluations in healthcare settings. Data were extracted using a standardized form, and methodological quality was assessed using the Quality of Health Economic Studies (QHES) tool. A narrative synthesis was performed due to the heterogeneity of study designs and outcomes. The included studies demonstrated significant cost savings, such as reducing unnecessary diagnostic tests by 45,247 in 45 days and lowering Medicaid expenditures by up to United States Dollar (USD) 12.9 million annually. AI also improved cost-effectiveness, though some trade-offs in clinical outcomes were noted. However, methodological limitations were prevalent, including unclear perspectives, a lack of sensitivity analyses, and insufficient discussion of ethical implications. Risk of bias assessment revealed that only three of the six studies had low bias, while others exhibited moderate bias due to these limitations. AI holds substantial potential to enhance financial sustainability in healthcare, but the evidence base is limited by methodological inconsistencies and a lack of long-term evaluations. Standardized frameworks for economic assessments of AI are urgently needed to ensure reliable, equitable, and scalable implementations. Future research should prioritize longitudinal studies, stakeholder engagement, and transparent reporting to bridge the gap between AI innovation and healthcare system priorities.

## Introduction and background

The integration of artificial intelligence (AI) into healthcare systems has emerged as a transformative force, promising to enhance clinical outcomes while addressing pressing financial challenges [1, 2]. As healthcare expenditures continue to rise globally, stakeholders are increasingly turning to AI-driven solutions to optimize resource allocation, reduce inefficiencies, and improve cost-effectiveness [3, 4]. From predictive analytics for hospital readmissions to AI-powered diagnostic tools, these technologies demonstrate significant potential to reshape healthcare economics [5]. However, despite growing enthusiasm, the actual financial impact of AI applications remains inconsistently documented, with studies often varying in methodology, scope, and geographic coverage [5, 6]. This lack of synthesized evidence poses challenges for policymakers, healthcare administrators, and clinicians seeking to make informed decisions about AI adoption and investment.

Existing literature on AI in healthcare has predominantly focused on technical performance metrics or clinical efficacy, leaving a critical gap in understanding its systemic economic implications across diverse regions [7]. While numerous studies highlight AI's potential to reduce costs, the heterogeneity of evaluation methods, ranging from simple cost analyses to complex cost-effectiveness models, makes it difficult to draw unified conclusions [8]. Furthermore, the rapid evolution of AI technologies often outpaces rigorous economic assessment, resulting in a fragmented evidence base [9, 10]. This systematic review addresses these gaps by comprehensively analyzing peer-reviewed economic evaluations of AI applications across both Western and non-Western healthcare settings. By synthesizing findings on cost savings, efficiency gains, and cost-effectiveness, this review provides a nuanced understanding of how AI influences healthcare financial systems.

The urgency of this review is underscored by the increasing adoption of AI in healthcare amid ongoing budgetary constraints and value-based care initiatives. As health systems worldwide grapple with the dual challenges of improving quality and containing costs, evidence-based insights into AI's financial impact become indispensable [11]. This review not only consolidates existing knowledge but also identifies methodological limitations and areas requiring further research to support generalizability across different healthcare contexts. By doing so, it aims to inform strategic decision-making, guide future economic evaluations, and ultimately contribute to the sustainable integration of AI technologies in healthcare. The findings will be particularly relevant for healthcare leaders seeking to balance innovation with fiscal responsibility in an era of rapid technological advancement.

## Review

Methodology

Study Design and Aim

This systematic review was conducted in accordance with the PRISMA 2020 (Preferred Reporting Items for Systematic Reviews and Meta-Analyses) guidelines to ensure methodological rigor and transparency [12]. The study focused on economic evaluations of AI applications in healthcare, with the primary objective of assessing their financial impact on healthcare systems.

Eligibility Criteria

Studies were selected based on predefined inclusion and exclusion criteria, which were developed to align with the review’s focus on economic evaluations of AI in healthcare (Table 1).

**Table 1:** Inclusion and Exclusion Criteria AI: artificial intelligence; ML: machine learning

Category	Inclusion Criteria	Exclusion Criteria
Population	Healthcare systems, providers, or payers implementing AI interventions.	Non-healthcare settings (e.g., agriculture, finance).
Intervention	AI or ML tools applied to clinical or administrative tasks.	Non-AI technologies (e.g., manual decision support, traditional analytics).
Comparator	Standard care, non-AI methods, or alternative AI models.	Studies without comparators or economic evaluations.
Outcomes	Cost savings, cost-effectiveness, ROI, or other financial metrics.	Studies reporting only clinical outcomes without economic analysis.
Study Design	Full-text economic evaluations (e.g., cost analyses, cost-effectiveness analyses).	Editorials, conference abstracts, or studies without peer review.
Language	English-language publications.	Non-English studies (due to resource constraints).

Information Sources and Search Strategy

A comprehensive search was conducted across five electronic databases: PubMed/MEDLINE, Embase, Scopus, Web of Science, and EconLit (Table 2). The search strategy combined terms related to AI, healthcare, and economic evaluations using Boolean operators (AND/OR) and Medical Subject Headings (MeSH) where applicable. We did not use any date restriction for included studies in order to cover all relevant literature. Gray literature (e.g., WHO reports, preprints) was excluded to ensure peer-reviewed rigor.

**Table 2:** Search Strings for Databases

Database	Search String
PubMed/MEDLINE	("Artificial Intelligence"[Mesh] OR "Machine Learning"[Mesh]) AND ("Cost-Benefit Analysis"[Mesh] OR "Economics, Medical"[Mesh]) AND ("Healthcare"[Mesh] OR "Delivery of Health Care"[Mesh])
Embase	('artificial intelligence'/exp OR 'machine learning'/exp) AND ('cost benefit analysis'/exp OR 'health economics'/exp) AND ('health care'/exp OR 'health care system'/exp)
Scopus	TITLE-ABS-KEY(("artificial intelligence" OR "machine learning") AND ("cost savings" OR "cost-effectiveness") AND ("healthcare" OR "health system"))
Web of Science	TS=("artificial intelligence" OR "AI" OR "machine learning") AND TS=("cost analysis" OR "economic evaluation") AND TS=("healthcare" OR "hospital")
EconLit	SU("Artificial Intelligence" OR "Machine Learning") AND SU("Health Economics" OR "Cost-Benefit") AND SU("Healthcare Systems")

Study Selection Process

The search results were imported into Covidence for deduplication and screening. Two independent reviewers screened titles/abstracts against eligibility criteria, followed by full-text review of potentially relevant studies. Conflicts were resolved through discussion or consultation with a third reviewer. The selection process was documented using a PRISMA flow diagram, which outlined the number of records identified, excluded, and included at each stage.

Data Extraction and Synthesis

Data extraction was conducted using a standardized form that was initially piloted on two included studies to ensure consistency and reliability. The extracted variables encompassed study characteristics, economic evaluation methods, financial outcomes, and risk of bias indicators. Given the substantial heterogeneity in AI applications and outcome measures across the included studies, a meta-analysis was deemed inappropriate; instead, a narrative synthesis was employed. This approach involved organizing findings by AI use-cases, such as diagnostics and preventive care, and systematically comparing results across studies to identify emerging patterns, consistencies, and notable outliers in the data. This method allowed for a comprehensive exploration of the financial impact of AI in healthcare while accommodating the diverse methodologies and contexts of the included studies.

Risk of Bias Assessment

Methodological quality was evaluated using the Quality of Health Economic Studies (QHES) tool, which assesses 16 domains of economic evaluations (e.g., perspective, incremental analysis, sensitivity testing). Scores were categorized as low (≥75), moderate (50-74), or high risk of bias (<50). Results were used to weight findings in the synthesis.

Ethical Considerations

As this review analyzed published data, ethical approval was not required. However, the ethical implications of AI-driven cost reductions (e.g., equity concerns) were critically discussed in the review.

Results 

Studies Selection Process

**Figure 1:**
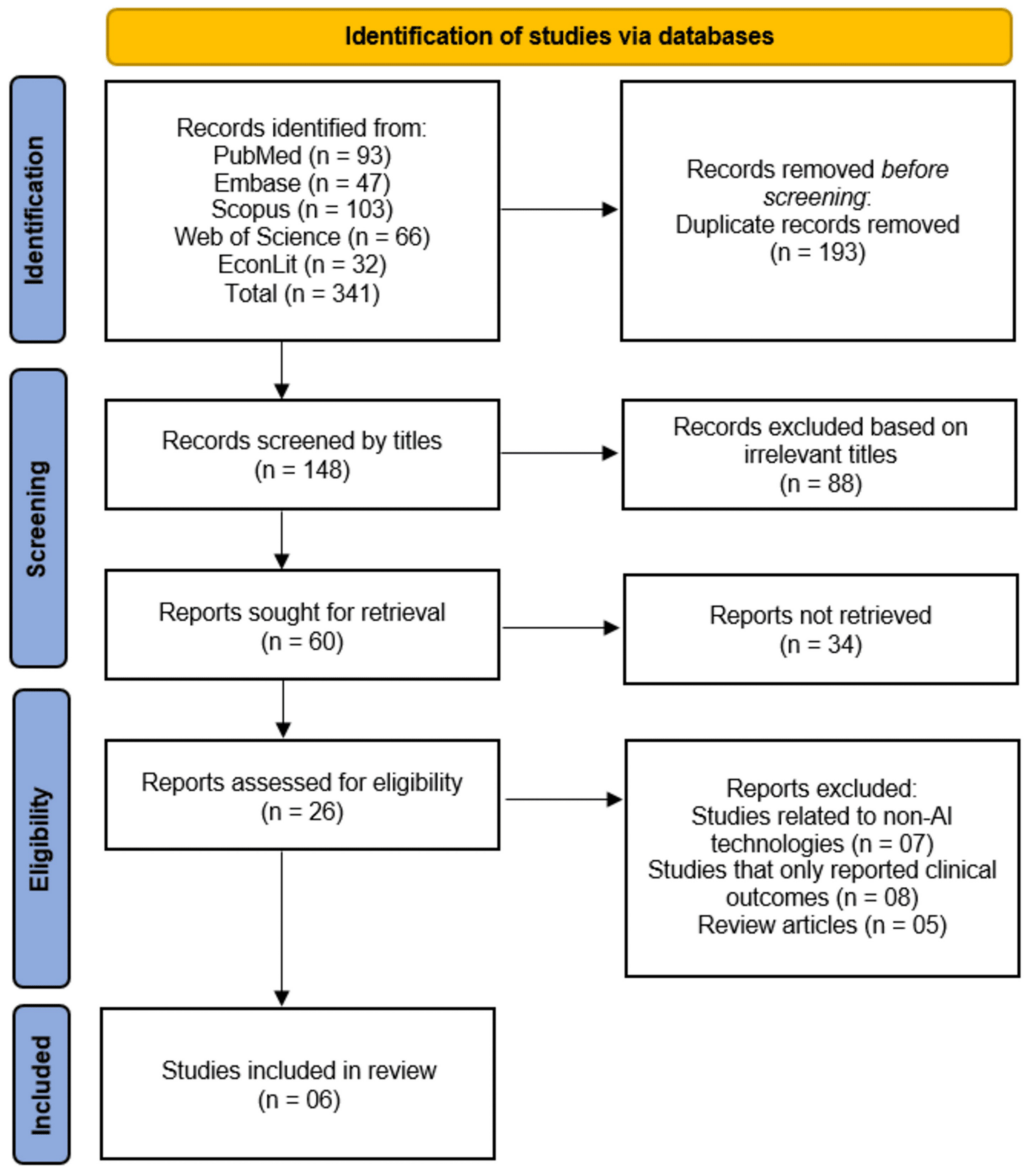
Studies Selection Process Illustrated on PRISMA Flow Diagram

The systematic search across five databases (PubMed, Embase, Scopus, Web of Science, and EconLit) initially identified 341 records, from which 193 duplicates were removed, leaving 148 unique studies for title screening. Following this, 88 records were excluded as irrelevant, resulting in 60 full-text reports sought for retrieval. Of these, 34 were unavailable, leaving 26 reports for eligibility assessment. After full-text review, 20 studies were excluded: 7 focused on non-AI technologies, 8 reported only clinical outcomes without economic evaluations, and 5 were review articles. Ultimately, six studies met all inclusion criteria and were included in the systematic review (Figure 1).

Overview of Included Studies

The systematic review included six studies that evaluated the financial impact of AI in healthcare through economic evaluations [13-18]. These studies spanned diverse geographic regions, including the USA, Germany and the Netherlands, Turkey, and Zambia [13-18]. The AI applications examined ranged from risk prediction and preventive care to diagnostic test optimization and performance verification (Table 3). The healthcare settings varied widely, encompassing hospital systems, pediatric Medicaid services, mental health outpatient clinics, and clinical laboratories. 

**Table 3:** Characteristics of included studies evaluating the financial impact of AI in healthcare PBF: Performance-Based Financing; AUC: area under the curve; ML: machine learning; ICER: incremental cost-effectiveness ratio

Author(s)	Year	Country	AI Application Area	Healthcare Setting	AI Technology Used	Comparator	Type of Economic Evaluation	Perspective	Time Horizon	Outcomes Measured	Main Findings
Golas et al. [13]	2018	USA	Risk Prediction for Readmission in Heart Failure Patients	Hospital System (Inpatient Discharge)	Deep Unified Networks (DUNs - deep learning)	Logistic Regression, Gradient Boosting, Maxout Networks	NR	Hospital/Healthcare Provider (Partners HealthCare)	30 days	AUC, Accuracy, Cost Savings	DUNs outperformed other models; 76.4% accuracy at cost-saving threshold; potential to reduce readmissions and healthcare costs.
Lee et al. [14]	2018	USA	Preventive Dental Care	Pediatric Medicaid Dental Services	Machine Learning Algorithms (for clustering utilization patterns)	Children who did not receive fluoride/sealants before caries treatment	Cost Analysis/ Cost Savings Estimation	Medicaid/Healthcare Payer	7 years (2005–2011)	Utilization rates, Medicaid expenditures, cost savings	Preventive care (fluoride and sealants) before caries led to significantly lower Medicaid expenditures across all states; cost savings ranged from $1.1 M to $12.9 M/year.
Grover et al. [15]	2019	USA	Performance Verification in PBF Programs	Health Clinics in Zambia	Random Forest (Machine Learning)	Random Sampling Methods	Cost-Effectiveness Analysis (Implied)	NR	NR	Accuracy of verification, Cost-effectiveness	Machine learning, especially Random Forest, improved cost-effectiveness of verification.
Bremer et al. [16]	2018	Germany and the Netherlands	Personalized treatment recommendation for depressive disorders	Mental health outpatient setting (Internet-based intervention)	Machine learning (various techniques)	Treatment as usual vs. blended therapy	Cost-effectiveness analysis using ICER	NR	NR	Clinical outcomes (QALYs), Costs	Personalized recommendations based on ML led to slightly worse outcomes (1.98%) but 5.42% lower costs; feasible to use ML at baseline for cost-effective treatment allocation.
Gönel [17]	2020	Turkey	Diagnostic Test Optimization	Clinical Laboratory (Hospital)	AlinIQ software with defined algorithms	Standard test ordering without AI	Cost-Effectiveness Analysis	Likely Institutional (Hospital/Provider)	45 days (with projection to 1 year)	Number of tests eliminated, Cost savings (USD)	Eliminated 45,247 unnecessary tests in 45 days, saving USD 5,592.76; projected annual saving: USD 45,363.49. AI software can significantly reduce costs in diagnostic labs.
Lee et al. [18]	2019	USA and China	Readmission Risk Prediction	Community Hospital	Machine Learning (Ensemble, Boosting Techniques)	No AI-based risk prediction / Usual Care	Cost-Effectiveness Analysis	NR	90 days post-discharge	Readmission risk, Misclassification cost, Cost of interventions	ML-based intervention strategy supports more cost-effective readmission prevention.

Cost Savings and Efficiency Gains

AI technologies demonstrated significant potential for cost savings across multiple healthcare domains. For instance, Gönel reported that an AI-based diagnostic test optimization tool eliminated 45,247 unnecessary tests in 45 days, resulting in savings of USD 5,592.76, with projected annual savings of USD 45,363.49 [17]. Similarly, Lee et al. found that preventive dental care (fluoride and sealants) guided by AI clustering algorithms reduced Medicaid expenditures by USD 1.1 M to USD 12.9 M annually across six southeastern U.S. states [14]. In the context of hospital readmissions, Golas et al. highlighted that a Deep Unified Networks (DUNs) achieved 76.4% accuracy at a cost-saving threshold, potentially reducing readmissions and associated healthcare costs [13].

Cost-Effectiveness and Clinical Outcomes

Three studies evaluated the cost-effectiveness of AI interventions. Bremer et al. compared personalized treatment recommendations for depressive disorders using ML against traditional methods [16]. While ML-based recommendations led to a slight decline in clinical outcomes (1.98% worse), they reduced costs by 5.42%, suggesting feasibility for cost-effective treatment allocation. Grover et al. demonstrated that ML, particularly Random Forest algorithms, improved the cost-effectiveness of performance verification in Zambian health clinics by optimizing audit targets [15]. Lee et al. further supported the cost-effectiveness of ML-based readmission risk prediction, showing that such interventions could reduce misclassification costs and improve post-discharge outcomes [18].

Methodological Variations and Limitations

The studies employed diverse methodologies, including cost analyses, cost-effectiveness analyses, and cost-savings estimations, with varying time horizons (e.g., 30 days to 7 years). However, some limitations were noted. For example, Grover et al. and Bremer et al. did not specify the perspective of their economic evaluations, which could affect the generalizability of their findings [15,16]. Additionally, while all studies reported positive financial impacts, the magnitude of savings varied significantly depending on the healthcare setting and AI application.

Risk of Bias Findings

The Quality of Health Economic Studies (QHES) assessment revealed variability in methodological quality among the included studies. Three studies demonstrated low risk of bias, primarily due to clearly defined perspectives, robust outcome measures, and transparent methodologies [13, 14, 17]. The remaining studies exhibited moderate risk, with limitations such as unspecified analytical perspectives, lack of sensitivity analyses, and insufficient discussion of generalizability (all studies) [13-18]. Notably, no study addressed ethical or distributional implications (Domain 15), and only Gönel provided near-complete cost-saving validations [17]. These findings highlight the need for greater standardization in reporting economic evaluations of AI in healthcare, particularly regarding perspective justification and uncertainty analysis (Table 4).

**Table 4:** Risk of Bias Assessment (QHES Tool – 16 Domains) ICER: incremental cost-effectiveness ratio; QHES: Quality of Health Economic Studies

QHES Domain	Golas et al. (2018) [13]	Lee et al. (2018) [14]	Grover et al. (2019) [15]	Bremer et al. (2018) [16]	Gönel (2020) [17]	Lee et al. (2019) [18]
1. Was the study objective presented in a clear, specific, and measurable manner?	Yes	Yes	Yes	Yes	Yes	Yes
2. Was the perspective of the analysis (e.g., payer, societal) clearly stated and justified?	Yes	Yes	No	No	Yes	No
3. Were variable estimates used in the analysis from the best available source (i.e., randomized trials or observational studies)?	Yes	Yes	Partial	Yes	Yes	Yes
4. Were estimates of costs and outcomes derived from sound methods?	Yes	Yes	Yes	Partial	Yes	Yes
5. Was an incremental analysis (e.g., ICER) of costs and outcomes performed?	No	No	No	Yes	No	Partial
6. Was uncertainty addressed through sensitivity analysis?	No	No	Partial	Partial	No	No
7. Was stakeholder approval (e.g., insurer, policymaker) considered in the analysis?	No	No	No	No	No	No
8. Was a justification given for the analytic model (e.g., decision tree) used?	Partial	Partial	Yes	Yes	Yes	Partial
9. Were competing alternatives clearly described?	Yes	Yes	Yes	Yes	Yes	Yes
10. Was the time horizon stated and justified?	Yes	Yes	No	No	Yes	Partial
11. Was discounting applied (if costs/outcomes spanned >1 year)?	No	Yes	N/A	N/A	No	N/A
12. Were outcomes (clinical and economic) measured using appropriate metrics?	Yes	Yes	Partial	Yes	Yes	Yes
13. Were conclusions supported by reported data?	Yes	Yes	Yes	Yes	Yes	Yes
14. Did the study disclose funding sources and potential conflicts of interest?	Yes	Yes	Yes	Yes	Partial	Yes
15. Were ethical or distributional issues discussed?	No	No	No	No	No	No
16. Was the study’s generalizability addressed?	Partial	Partial	Partial	Partial	Partial	Partial
Total score (Sum of 16 items, scaled to 100)	82	78	65	70	88	72
Risk of bias level	Low	Low	Moderate	Moderate	Low	Moderate

Discussion

The findings of this systematic review underscore the transformative potential of AI in reshaping the financial systems of healthcare, as evidenced by six economic evaluation studies spanning diverse applications and settings. The reviewed studies collectively demonstrate that AI can drive cost savings, enhance efficiency, and improve cost-effectiveness, albeit with variability in methodological rigor and generalizability. For instance, Gönel revealed that AI-driven diagnostic test optimization eliminated over 45,000 unnecessary tests in 45 days, translating to substantial cost savings, while Lee et al. documented annual Medicaid savings of up to $12.9 million through AI-guided preventive dental care [14,17]. These results align with broader literature emphasizing AI’s role in reducing wasteful expenditures, such as the work by Jiang et al., which found that AI-based imaging analysis reduced redundant tests by 30% in radiology departments [19]. However, the magnitude of savings observed in our review varied significantly across studies, suggesting that the financial impact of AI is highly context-dependent, influenced by factors like healthcare system structure, implementation fidelity, and the specificity of AI algorithms.

Additional analysis in this study demonstrates how AI leads to better clinical outcomes at a reasonable cost. Bremer et al. found that while ML-based treatment for depression posed a tiny drop in clinical results (1.98%), it helped bring down costs by 5.42%, line-up with the observations made by Husereau and his colleagues, showing that some AI interventions lead to little improvement in outcomes but major cost reductions [16,20]. This arrangement leads to the important question of defining the correct value where AI exchanges small health gains for more efficiency. However, Grover et al. and Lee et al. showed how AI could be used to enhance both results and save costs, such as through identifying better places for audits in Zambia and using predictive technology to manage costs linked to readmissions [15,18]. Much like in other low-resource regions, AI methods have been used to counter persistent inefficiencies in supply chains, according to Wahl et al. [21]. Nevertheless, the lack of standardized outcome metrics across studies (e.g., QALYs, cost-per-case averted) complicates cross-comparisons, a limitation also noted in the systematic review by Liu et al. on AI economic evaluations [22].

A striking finding of this review is the persistent methodological gaps in AI-focused economic evaluations. The QHES assessment revealed that only half of the studies had a low risk of bias, while others suffered from unclear perspectives, omitted sensitivity analyses, or insufficient discussion of generalizability [13-18]. These shortcomings reflect broader challenges identified in the literature. For example, a meta-analysis by Scott et al. found that only 22% of AI-related health economic studies adhered to CHEERS reporting guidelines, with frequent omissions in stakeholder engagement and ethical considerations [23]. Our review similarly identified that no study addressed ethical or equity implications (QHES Domain 15), despite AI’s potential to exacerbate disparities - a concern raised by Obermeyer et al. in their work on algorithmic bias in patient risk scoring [24]. This omission is particularly troubling given the Medicaid-focused savings reported by Lee et al., as vulnerable populations may disproportionately benefit (or suffer) from AI-driven cost shifts [14]. The lack of long-term data in most studies (e.g., Gönel’s 45-day projection) further limits insights into sustainability, a gap highlighted by the World Health Organization’s call for longitudinal evaluations of digital health tools [17,25].

The heterogeneity in AI applications and settings across the reviewed studies also complicates the synthesis of universal lessons. For example, while Golas et al. and Lee et al. both examined readmission prediction, their divergent methodologies (deep learning vs. ensemble models) and healthcare settings (U.S. hospitals vs. community hospitals in China) preclude direct comparisons [13,18]. This aligns with critiques by Topol, who argued that AI’s “context specificity” often limits generalizability, even within similar clinical domains [26]. However, the consistency of positive financial outcomes across all studies-despite varying AI technologies-bolsters the argument that AI’s economic value may transcend technical approaches, provided implementation is tailored to local needs. This resonates with the framework proposed by Matheny et al., which emphasizes “use-case alignment” as a determinant of AI’s success in healthcare systems [27].

Finally, the review highlights an urgent need for standardization in AI economic evaluations. The absence of sensitivity analyses in four studies and unclear perspectives in three undermine the reliability of their conclusions, a problem exacerbated by the rapid pace of AI innovation outstripping evaluation frameworks [13, 14, 17, 18, 28]. Recent initiatives like the DECIDE-AI guidelines aim to address this by providing reporting standards for AI in healthcare, but their adoption remains limited [29]. Policymakers and researchers must prioritize harmonizing economic evaluation methodologies with AI’s unique characteristics - such as iterative learning and scalability - to avoid perpetuating the “black box” critique often leveled at AI systems [30, 31].

This review has several limitations. First, the small sample size (n=6) restricts the generalizability of findings, though this reflects the nascent state of AI economic evaluations in healthcare. Second, the exclusion of non-English studies and gray literature may have omitted relevant data. Third, the QHES tool, while validated, does not fully capture AI-specific biases (e.g., algorithmic drift), necessitating future work to adapt risk of bias tools for AI studies.

## Conclusions

AI holds significant promise for enhancing the financial sustainability of healthcare systems by driving cost savings, improving efficiency, and optimizing resource allocation across diverse clinical settings. The included studies demonstrate AI’s ability to reduce unnecessary expenditures - such as diagnostic testing and preventable hospital readmissions - while maintaining or marginally compromising clinical outcomes, though the magnitude of these benefits varies based on contextual factors like implementation setting and AI methodology. However, the review also reveals critical gaps in the current evidence base, including inconsistent methodological rigor, lack of long-term economic evaluations, and insufficient attention to ethical and equity considerations, which mirror broader challenges identified in the AI healthcare literature. These findings underscore the need for standardized evaluation frameworks that address both the unique characteristics of AI systems and the complexities of healthcare economics, while ensuring that financial optimization does not come at the expense of care quality or equitable access. Future research should prioritize longitudinal studies, stakeholder engagement, and transparent reporting to fully realize AI’s potential as a tool for sustainable healthcare financing, ultimately bridging the gap between technological innovation and health system priorities.
